# Association between Inflammatory Bowel Disease and Pancreatitis: A PRISMA-Compliant Systematic Review

**DOI:** 10.1155/2020/7305241

**Published:** 2020-08-03

**Authors:** Pengfan Li, Kanjun Chen, Zheng Mao, Yue Luo, Yan Xue, Yuli Zhang, Xueying Wang, Lihang Zhang, Sizhen Gu, Danbo Dou

**Affiliations:** ^1^Department of Traditional Chinese Medicine, Shuguang Hospital Affiliated to Shanghai University of Traditional Chinese Medicine, Shanghai 201203, China; ^2^Department of Foreign Language Teaching Center, Shanghai University of Traditional Chinese Medicine, Shanghai 201203, China; ^3^Department of Dermatology, Yueyang Hospital of Integrated Traditional Chinese and Western Medicine, Shanghai University of Traditional Chinese Medicine, Shanghai 200437, China; ^4^Institute of Tramotology and Orthopedics, Shuguang Hospital Affiliated to Shanghai University of Traditional Chinese Medicine, Shanghai 201203, China

## Abstract

**Methods:**

MEDLINE, Embase, and CENTRAL were systematically searched for correlative studies till 2 November 2019. RevMan5.3 was used to estimate relevance.

**Results:**

Three studies with 166008 participants were included. The risk of pancreatitis significantly increased in the patients with CD (OR, 3.40; 95% CI, 2.70-4.28; *P* < 0.00001) and UC (OR, 2.49; 95% CI, 1.91-3.26; *P* < 0.00001). Increased risks of CD (OR, 12.90; 95% CI, 5.15-32.50; *P* < 0.00001) and UC (OR, 2.80; 95% CI, 1.00-7.86; *P* = 0.05) were found in patients with chronic pancreatitis. As for patients with acute pancreatitis, there were significant association of CD (OR, 3.70; 95% CI, 1.90-7.60; *P* = 0.0002), but were not UC.

**Conclusions:**

The evidence confirmed an association between pancreatitis and IBD. When pancreatitis patients have chronic diarrhea and mucus blood stool or IBD patients have repeated abdominal pain and weight loss, they should consult pancreatic and gastrointestinal specialists.

## 1. Introduction

Pancreatitis is a kind of pancreatic inflammatory injury caused by hyperaemia, edema, haemorrhage, and necrosis, which leads to the self-digestion of pancreatic tissue [1]. Acute pancreatitis (AP) is a type of commonest clinical acute abdomen, which may endanger the life of patients [2]. Despite extensive research and the rapid development of medicine in the past decade, the mortality rates of AP are still high around the world [3]. Additionally, about 18% of patients with AP recurred and 8% developed to chronic pancreatitis (CP) [4, 5]. The gastrointestinal disorder is a common clinical phenomenon in pancreatitis [6]. The causes are diverse, mostly related to immune abnormalities, microcirculatory disorders, genetic susceptibility, dehydration, malnutrition, enterobacter with the ability to produce amylase, and the excretion of intestinal amylase into the blood [7].

Inflammatory bowel disease (IBD), a type of chronic recurrent alimentary canal disease characterized by abdominal pain, diarrhea with bloody purulent stool or mucus, and tenesmus, consists of 2 predominant types: ulcerative colitis (UC) and Crohn disease (CD) [8]. In the last 20 years, the incidence rate of IBD in developing countries has been rising rapidly, with an annual increase rate of 11.1% (95% CI 4.8-17.8) for CD and 14.9% (95% CI 10.4-19.6) for UC [9]. Increasing research evidence suggests that the genetic susceptibility to inflammatory response disorders and microbiota changes may play a momentous role in the pathomechanism of IBD [10–12]. UC mainly involves invasion of the mucous layer and submucosa of the colon and rectum, while CD frequently leads to invasion and damage of all parietal layers along the alimentary canal [11].

Pancreatitis shares common clinical manifestations, genetic susceptibility, microflora alteration, and immunologic features with IBD. Various types of pancreatitis may occur in patients with IBD due to the disease itself or side effects of medication used in the treatment [13]. Both diseases have similar clinical manifestations, such as abdominal pain, abdominal distension, anorexia, fever, diarrhea, and vomiting [8, 14–16]. Although some studies have shown that there is a link between pancreatitis and IBD [15, 17, 18], data on this link remain inconsistent and unclear. For instance, a previous study failed to detect a significant increase of UC in patients with pancreatitis [19]. In this study, we aim to systematically and comprehensively examine the evidence of pancreatitis associated with IBD.

## 2. Methods

### 2.1. Eligibility Criteria

According to the Preferred Reporting Items for Systematic Reviews and Meta-analyses (PRISMA) [20] and Meta-analysis of Observational Studies in Epidemiology (MOOSE) guidelines [21], we conducted a systematic review of observational researches on the relevance between pancreatitis and IBD. It has been registered with PROSPERO (CRD42020156756). And the PRISMA and MOOSE guidelines are shown in the Supplementary Materials (available here).

### 2.2. Evidence Search

The types of eligible literatures included cross-sectional, case-control, and cohort studies. The MEDLINE, Cochrane Central Register of Controlled Trials (CENTRAL), and Embase databases were retrieved for relevant studies from the respective inception of these databases to 2 November 2019. No linguistic or geographic restrictions were imposed. The detailed search strategy was listed in Table SM1 in the Supplementary Materials.

### 2.3. Selection of Studies

Studies that met the following inclusion criteria were included: (1) observational researches investigated the relevance between pancreatitis and IBD, including cross-sectional, case-control, or cohort studies; (2) human research participants; and (3) the case/exposed group was made up of patients with pancreatitis, and the control group was made up of people without pancreatitis. Or the case/exposed group was IBD patients, and the control group was made up of people without IBD. Three authors (Pengfan Li, Kanjun Chen, and Zheng Mao) screened relevant researches independently by scanning titles and abstracts. Four authors (Pengfan Li, Yue Luo, Yan Xue, and Sizhen Gu) reviewed the full story of included studies and potentially eligible researches which met the inclusion criteria. Disagreement got the solution by discussion.

### 2.4. Data Extraction and Risk of Bias Assessment

Two authors (Yuli Zhang and Xueying Wang) extracted the following data from the included literatures: study design, first author, publication year, country, and risk estimate, including odds ratio (OR) with corresponding 95% CIs about the association between pancreatitis and IBD. When using hazard ratio (HR) for risk estimation, we convert it to OR [22, 23]. The Newcastle-Ottawa Scale (NOS) was utilized for assessing the bias of included researches [24]. Doubt or disagreement got the solution by contacting the original author or discussion.

### 2.5. Statistical Analysis

Through using the Review Manager software (version 5.3) [25], we computed an OR with 95% CI for included studies. And the random effects model was selected for this systematic review because of anticipated clinical heterogeneity [26].

## 3. Results

### 3.1. Characteristics of Included Studies

**Figure 1:**
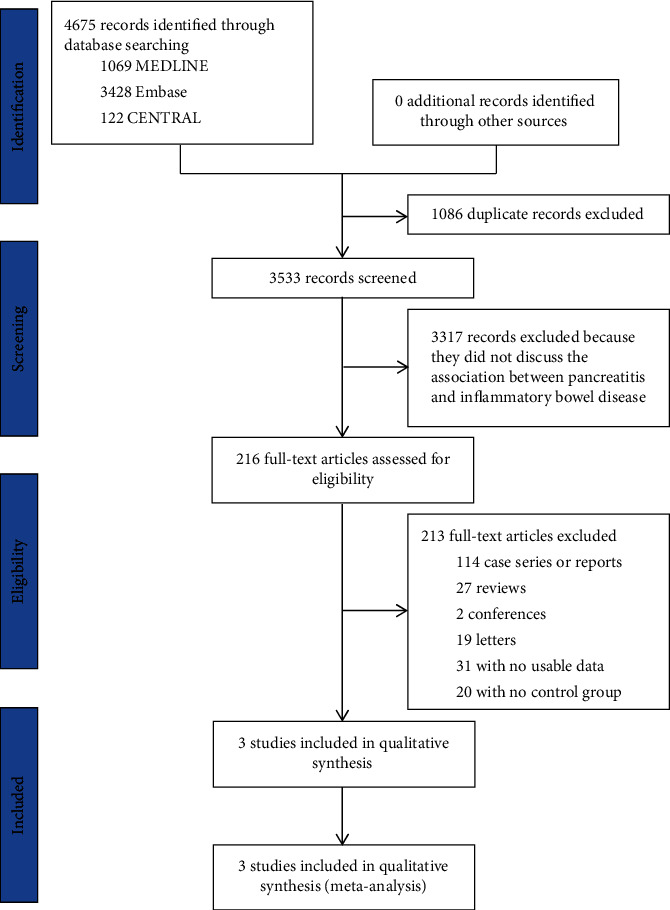
PRISMA study flowchart.

The PRISMA study flow diagram was shown in Figure 1. After duplicates excluded, 3533 records were identified by our search. We excluded 3317 literatures after scanning all the titles and abstracts. After detailed examination of the full texts, we included 2 case-control studies [19, 27] and 1 cohort study [28] with a total of 166008 unique study participants. One study was conducted in the west countries [19], and the other 2 were conducted in Asia [27, 28]. The characteristics of all the included studies are shown in Table 1.

### 3.2. Quality Assessment and Risk of Bias

**Figure 2:**
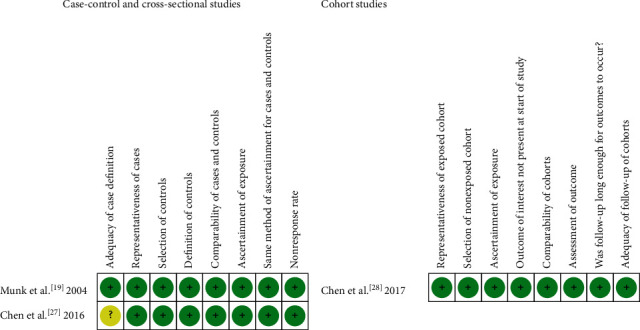
Risk of bias of included studies. High risk of bias (+); unclear risk of bias (?); low risk of bias (-).

The NOS quality assessment is shown in Table 1. All studies were greater than or equal to 8 points in NOS quality assessment, which means that all studies are of high quality. And the risk of bias among included studies is summarized in Figure 2. As to adequacy of case definition, only one study [27] was rated with an unclear risk because only codes from the International Classification of Diseases (8th, 9th, and 10th Revision) were used for identification of cases. The rest of the items not mentioned above, with reference to the NOS [24], were identified as a low risk of bias.

### 3.3. Association between Pancreatitis and IBD

**Figure 3:**
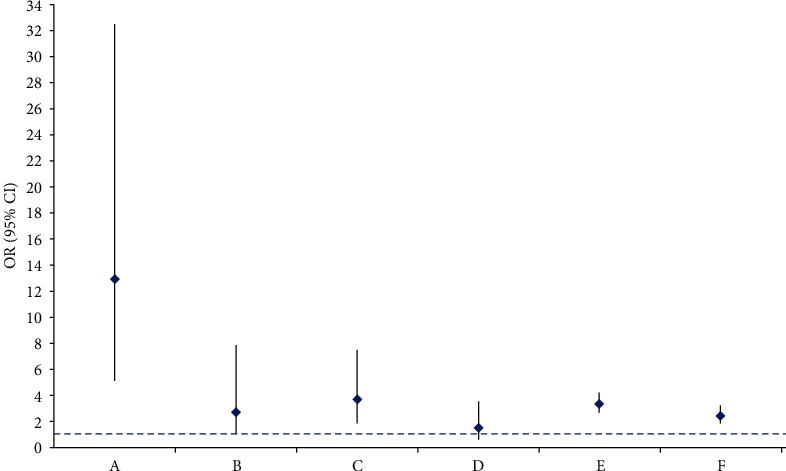
Association estimates between pancreatitis and inflammatory bowel disease. (a) The risk of Crohn disease in the patients with chronic pancreatitis; (b) the risk of ulcerative colitis in the patients with chronic pancreatitis; (c) the risk of Crohn disease in the patients with acute pancreatitis; (d) the risk of ulcerative colitis in the patients with acute pancreatitis; (e) the risk of acute pancreatitis in the patients with Crohn disease; (f) the risk of acute pancreatitis in the patients with ulcerative colitis.

As illustrated in Table 2 and Figure 3, one cohort study [28] with 17796 study participants found an increased risks of CD (OR, 12.90; 95% CI, 5.15-32.50; *P* < 0.00001) and UC (OR, 2.80; 95% CI, 1.00-7.86; *P* = 0.05) in patients with CP. One case-control study [19] with 1590 study participants indicated a conspicuously increased risk of CD in patients with AP (OR, 3.70; 95% CI, 1.90-7.60; *P* = 0.0002). However, the risk of UC did not increase markedly in patients with pancreatitis (OR, 1.50; 95% CI, 0.70-3.60; *P* = 0.32). And one case-control study [27] with 11909 study participants illustrated a noteworthy increased risk of acute pancreatitis in the patients with UC and CD. The results proved that the risk of acute pancreatitis significantly increased in the patients with CD (OR, 3.40; 95% CI, 2.70-4.28; *P* < 0.00001) and UC (OR, 2.49; 95% CI, 1.91-3.26; *P* < 0.00001).

## 4. Discussion

To our best knowledge, this study is the first systematic review which gathered all available data to evaluate the association between pancreatitis and IBD. We confirmed that IBD patients were inclined to have comorbid pancreatitis. Meanwhile, patients with pancreatitis were inclined to comorbid CD, but were not prone to comorbid UC. The evidence about the cohort study demonstrated that patients with pancreatitis had 12.9-fold OR of CD and 2.80-fold OR of UC when compared with control group. Meanwhile, the indication from the case-control study testified that those patients with pancreatitis had 3.7-fold OR of CD. As for patients with IBD, the indication from case-control study testified that those patients with CD had 3.4-fold OR of pancreatitis, and patients with UC had 2.49-fold OR of pancreatitis when compared with control group.

Pancreatitis and IBD share many characteristics, including clinical manifestations, genetic susceptibility, microflora alteration, and immunologic features. There are some explanations of the link between pancreatitis and IBD. Firstly, genetic susceptibility loci shared by pancreatitis and IBD have been found. Certain genes, such as the myosin IXB (MYO9B) gene and its two close-connected adaptor genes, MAGI2 and PARD3, have been associated with pancreatitis as well as IBD [29–31]. Secondly, emerging studies have shown that pancreatitis and IBD are diseases of immune dysregulation. Cytokine abnormalities, such as elevation of interleukin 1*β* (IL-1*β*), IL-6, IL-8, and IL-10, are involved in pancreatitis and IBD [32–34]. Pancreatic tissue and gastrointestinal epithelial cells may share vulnerable cellular structures or target molecules [35]. It had been proved that T-cells carrying MUC1 mucin migrated to the colon and pancreas at one time in IBD mice [36]. Experimental data obtained from trinitrobenzene sulfonic acid-induced colitis in mice testified simultaneous pancreatic injury [37]. And some researches indicated pancreatic antibodies in the serum of IBD patients associated with pancreatitis [38, 39]. Although the relevance between these antibodies and IBD and the pathomechanism of pancreatitis is not clear, those antibodies may reflect the immune imbalance partly between pancreatitis and IBD [40]. Thirdly, the alteration of microflora of immune response disorders may play a very momentous role in pancreatitis and IBD [41, 42]. Microflora can recognize pathogens and repair damage by activating Toll-like receptors, thus affecting the immune and physiological homeostasis of pancreatic tissue and intestinal mucosa [42–44]. However, all kinds of environmental factors can change the microbial balance, resulting in the reduction of microbial diversity [1]. This change in microflora may lead to immune disorders and susceptibility to disease, including pancreatitis and IBD [45]. Studies have shown that whether patients have acute pancreatitis or chronic pancreatitis, their levels of *Bifidobacterium* or *Lactobacillus* are lower and levels of *Enterobacteriaceae* are higher [46, 47]. Increasing evidence indicates that the alterations of intestinal microbiota may be related to the pathomechanism of IBD, with decreases in specific *Firmicutes* species and increases in facultative anaerobes and *Bacteroidetes* species [48]. Changes of microbiota may lead to systemic immune impairment. Such a close interaction between changes of microbiota, cytokines, and pancreas has been considered as the gut-pancreas axis theory [49].

This study also has some limitations. Only 3 studies [19, 27, 28] that met the inclusion criteria have been included, because the publication bias was unclear. Meanwhile, the majority of included studies were from eastern countries (China) [27, 28], with only 1 from Europe (Denmark) [19]. More high-quality evidence is needed to confirm this result in the future. Nonetheless, the overall direction of results was identical.

## 5. Conclusions

Up to now, the evidence confirmed a noteworthy association between pancreatitis and IBD. Patients with pancreatitis should be told of an increased risk of IBD, and patients with IBD should also be told of an increased risk of pancreatitis. When pancreatitis patients have chronic diarrhea and mucus blood stool or IBD patients have repeated abdominal pain and weight loss, they should consult pancreatic and gastrointestinal specialists.

## Figures and Tables

**Table 1:** Characteristics of included studies.

Source	Country	Study design	Exposed group (cohort study)/case group (case-control study)	Control group	Odds ratio (CI)		Quality assessment (Newcastle-Ottawa scale)
					Ulcerative colitis	Crohn disease	
Munk et al. [19] 2004	Denmark	Case-control study	1590 patients with acute pancreatitis from the Hospital Discharge Registry of the North Jutland County of Denmark from 1991 to 2002 (830 male, 760 female)	15913 patients from the Central Personal Registry, matched by age and gender (8304 male, 7609 female)	1.50 (0.70-3.60)	3.70 (1.90-7.60)	Selection: 4
							Comparability: 2
							Exposure: 3
Chen et al. [27] 2016	China	Case-control study	11909 patients diagnosed with IBD between 2000 and 2010 from the National Health Insurance Research Database of Taiwan (6418 male, 5491 female)	A comparison cohort comprised 47636 age-matched patients without IBD (25672 male, 21964 female)	2.49 (1.91-3.26)	3.40 (2.70-4.28)	Selection: 3
							Comparability: 2
							Exposure: 3
Chen et al. [28] 2017	China	Cohort study	17796 patients with newly diagnosed chronic pancreatitis between 2000 and 2010 (14685 male, 3111 female)	71164 matched patients without chronic pancreatitis (58720 male, 12444 female)	2.80 (1.00-7.86)	12.9 (5.15-32.50)	Selection: 4
							Comparability: 1
							Outcome: 3

NHIS: National Health Information Service; IBD: inflammatory bowel disease; CI: confidence interval.

**Table 2:** Association between pancreatitis and inflammatory bowel disease.

Trails	Study design	Association estimates [95% CI]		*P* value
1. The risk of Crohn disease in the patients with chronic pancreatitis				
Chen 2017	Cohort study	OR	12.90 [5.15, 32.50]	<0.00001
2. The risk of ulcerative colitis in the patients with chronic pancreatitis.				
Chen 2017	Cohort study	OR	2.80 [1.00, 7.86]	0.05
3. The risk of Crohn disease in the patients with acute pancreatitis.				
Munk 2004	Case-control study	OR	3.70 [1.90, 7.60]	0.0002
4. The risk of ulcerative colitis in the patients with acute pancreatitis.				
Munk 2004	Case-control study	OR	1.50 [0.70, 3.60]	0.32
5. The risk of acute pancreatitis in the patients with Crohn disease.				
Chen 2016	Case-control study	OR	3.40 [2.70, 4.28]	<0.00001
6. The risk of acute pancreatitis in the patients with ulcerative colitis.				
Chen 2016	Case-control study	OR	2.49 [1.91, 3.26]	<0.00001

## Supplementary Materials

Supplementary Materials Table SM1: search strategy. Table SM2: PRISMA checklist. Table SM3: MOOSE checklist.
